# *Salmonella enterica* subsp. *enterica* serovar Paratyphi B from mute swan (*Cygnus olor*): complete genome sequence features point towards invasive variant potential

**DOI:** 10.1128/mra.01056-23

**Published:** 2024-05-29

**Authors:** Marina C. Lamparter, Maria Borowiak, Peter Kutzer, Patricia Schlieben, Istvan Szabo, Jennie Fischer

**Affiliations:** 1 Department of Biological Safety, Food Microbiology, Host-Pathogen-Interactions, German Federal Institute for Risk Assessment (BfR), Berlin, Germany; 2 Department of Biological Safety, National Study Centre for Sequencing in Risk Assessment, German Federal Institute for Risk Assessment (BfR), Berlin, Germany; 3 Landeslabor Berlin-Brandenburg, Frankfurt (Oder), Germany; University of Maryland School of Medicine, Baltimore, Maryland, USA

**Keywords:** *Salmonella enterica* subsp. *enterica* Paratyphi B, invasive, water bird, Germany

## Abstract

A subgroup of Salmonella (S.) enterica subsp. enterica serovar Paratyphi B is significantly associated with invasive infections in humans. We report the complete genome sequence of a potentially invasive. *S.* Paratyphi B isolated from a mute swan (*Cygnus olor*) found dead at an urban recreation park in Berlin, Germany.

## ANNOUNCEMENT

The non-typhoidal *d*-tartrate fermenting (*d*T+) variant of *S.* Paratyphi B (formerly variant Java) is common in poultry and patients in Europe. In contrast, the systemic, invasive, and non-*d*-tartrate fermenting (*d*T−) *S.* Paratyphi B variant is rarely reported from patients without travel history, and animal findings are, to our knowledge, not documented. We isolated a *S.* Paratyphi B closely related to the invasive biotype from an adult female mute swan found dead at an urban recreation park water side in Berlin, Germany. Pathological investigations confirmed myocarditis due to *Sarconema* sp. as the probable cause of death. *Salmonella* isolation was performed following the procedure for samples from the primary production stage given in ISO 6579-1:2017, using approximately 5 g of an organ pool (lung, liver, spleen) and intestinal content (1). Suspicious colonies were confirmed as *Salmonella* spp. using matrix-assisted laser desorption ionization-time of flight mass spectrometry (MALDI-TOF-MS) (Bruker Daltonics). Serological typing was performed according to the White-Kaufmann-Le Minor Scheme using standard reagents (Sifin diagnostics GmbH Berlin, Germany); *d*T variant PCR screening targeting the STM3356 Start codon was done according to (2, 3).

For isolation of genomic DNA (gDNA), one colony of isolate 22-SA01722-0 was enriched in lysogeny broth for 18 hours at 37 ± 1°C. One PureLink Genomic DNA mini kit gDNA isolation preparation (Invitrogen, Carlsbad, CA, USA) was used for short- and long-read sequencing.

The library for short-read sequencing was prepared using the Illumina DNA Prep (M) Tagmentation kit (Illumina, San Diego, CA, USA). The library was sequenced on the Illumina NextSeq benchtop sequencer using the NextSeq 500/550 MID output kit v2.5 (300 cycles, Illumina) in 2 × 149 bp cycles. The short-read sequence data were trimmed using fastp v0.22.0 (4). Trimming resulted in 2,361,633 high-quality reads (85%, quality score ≥Q30).

The library for the Oxford Nanopore MinION technology was prepared using the Rapid Barcoding kit SQK-RBK110.96 (Oxford Nanopore Technologies, Oxford, UK). Kits were used according to the manufacturer’s instructions.

The MinION library was sequenced for 72 h on an ONT MinION Mk1C device using a MinION Flowcell R9.4.1. The obtained fast5 data were basecalled on a high-performance GPU computer using guppy v6.0.1 in SUP mode (https://nanopore tech.com/community). The generated reads were trimmed using Porechop v0.2.4 (https://github.com/rrwick/Porechop), filtered using NanoFilt v2.8.0, and quality checked using NanoStat v1.5.0 (5). Trimming and filtering resulted in 17,069 reads with a read length N50 value of 14,972 bp and a mean quality of 13.6.

To assemble and circularize the genome sequence, short- and long-read data sets were subjected to the hybrid assembler Unicycler v0.4.8 including Pilon v1.24 (6–9).

The assembly resulted in a circular bacterial chromosome (start gene: *dnaA*). BakCharak pipeline v3.0.4 and PGAP v6.5 (annotation) were used for genome characterization (10–22). ChewieSnake pipeline and NCBI blastn were used for phylogenetic comparison to *d*T- Paratyphi B variant strains (18, 23–25). All tools, database versions, and parameters used for bioinformatics analyses and the results are summarized in Table 1. Default parameters were used unless otherwise specified. Results hint toward an invasive potential of isolate 22-SA01722-0.

**TABLE 1:** Summary of whole-genome-sequenced-based genetic features of *S*. Paratyphi B 22-SA01722-0 and comparison with *S*. Paratyphi B strains from Conner et al. (23)^*a*^

Features	Tools, databases and parameters	Results for isolate 22-SA01722-0	Features of invasive *S*. Paratyphi variant	References
Serovar	Sistr_cmd v1.1.1	*Salmonella enterica* subsp *enterica* serovar Paratyphi B		2, 15
Chromosome size—GC content	Geneious Prime v2020.2.2	4,761,312 bp - 53.8%		
MLST^*b*^	MLST v2.23.0 db PubMLST	ST86	ST86	13, 23
Virulence determinants	ABRicate v1.0.1, db VFDB_all_setA, minicov: 80, minid: 50 and db inhouse curated SPI (based on PAIdb, NCBI) minicov/minid: 30, v2020-09-03; *SPI-6: visualization of genome annotation (PGAP v6.5) in Geneious Prime v2020.2.2 and blastn nt alignment (*pagN* – tRNA-Asp; 43,161 bases) against ERR129894	*Salmonella* Pathogenicity Islands (SPIs) detected: SPI-1 to SPI-5, SPI-6*, SPI-9, SPI-11 to SPI-14 and SPI-16 Number of detected virulence genes: 151; assigned in functional categories: adherence: 25, Antimicrobial activity/competitive advantage: 2, effector delivery system: 108, immune modulation: 2, invasion: 2, nutritional/metabolic factor: 7, regulation: 5 SPI-6* blastn: 43,152/43,161 (99.98%) nt identity	SPI-1 to SPI-5, SPI-6*, SPI-9, SPI-11 to SPI-14, and SPI-16	10, 11, 12, 16, 17, 19–25
STM3356 (locus tag Q1K22_RS02410) start codon sequence	PGAP v6.5, visualization of genome annotation in Geneious Prime v2020.2.2	ATA	ATA	3, 19–21
Further genetic features associated with invasive disease phenotype	ABRicate v1.0.1, db VFDB_all_setA, minicov: 80, minid: 50; PGAP v6.5, visualization of genome annotation in Geneious Prime v2020.2.2	*sseJ* and *steB* absent	Absence of *sseJ* and *steB*	10, 11, 12, 19–23
22-SA01722-0 closest relatives of Connor et al. 2016 *S*. Paratyphi B selection	Aquamis v1.3.12, chewieSnake v3.2	Closest relatives from the Conner *et al*. 2016 study: ERR129894 (28 AD), ERR129896 (31 AD), and ERR023396 (29 AD), (≥97% cgMLST loci detected)	Closest relatives belong to phylo-group 1, all associated with invasive type/matrix	18, 23, 24

^*a*^ For bacterial characterization, Bakcharak v3.0.4 with implemented tools and PGAP v6.5 were used. For core genome multi locus sequence typing (cgMLST) genome comparison, chewieSnake v3.2 was used. For SPI-6 nt comparison, blastn (https://blast.ncbi.nlm.nih.gov/Blast.cgi) was additionally used. Default parameters were used for all tools unless otherwise specified.

^*b*^ MLST, multi locus sequence typing; cg, core genome; ST, sequence type; SPI, Salmonella pathogenicity islands; db, database; QC, quality control; AD, allelic distance; nt, nucleotide; blastn, basic local alignment search tool nucleotide.

## Data Availability

Sequencing raw reads were deposited in the NCBI Sequence Read Archive (SRA) (accession numbers SRR25476016 [ONT data] and SRR25476017 [Illumina data]). The complete genome sequence of 22-SA01722-0 is available at NCBI (GenBank sequence CP130564 [chromosome]). All data are encompassed under BioProject number PRJNA994055, BioSample SAMN36414958.

## References

[1] Anonymous. 2020. Microbiology of the food chain - horizontal method for the detection, enumeration and serotyping of Salmonella - part 1: detection of Salmonella spp. (ISO 6579-1:2017 + Amd.1:2020). Geneva International Organization for Standardization

[2] Grimont PA, Weill F-X. 2007. WHO collaborating centre for reference and research on *Salmonella*, p 1–166. In Antigenic formulae of the Salmonella serovars. Vol. 9.

[3] Malorny B, Bunge C, Helmuth R. 2003. Discrimination of d-tartrate-fermenting and -nonfermenting Salmonella enterica subsp. enterica isolates by genotypic and phenotypic methods. J Clin Microbiol 41:4292–4297. doi:10.1128/JCM.41.9.4292-4297.2003 12958259 PMC193836

[4] Chen S, Zhou Y, Chen Y, Gu J. 2018. Fastp: an ultra-fast all-in-one FASTQ preprocessor. Bioinformatics 34:i884–i890. doi:10.1093/bioinformatics/bty560 30423086 PMC6129281

[5] De Coster W, D’Hert S, Schultz DT, Cruts M, Van Broeckhoven C. 2018. Nanopack: visualizing and processing long-read sequencing data. Bioinformatics 34:2666–2669. doi:10.1093/bioinformatics/bty149 29547981 PMC6061794

[6] Walker BJ, Abeel T, Shea T, Priest M, Abouelliel A, Sakthikumar S, Cuomo CA, Zeng Q, Wortman J, Young SK, Earl AM. 2014. Pilon: an integrated tool for comprehensive microbial variant detection and genome assembly improvement. PLoS One 9:e112963. doi:10.1371/journal.pone.0112963 25409509 PMC4237348

[7] Wick RR, Judd LM, Gorrie CL, Holt KE. 2017. Unicycler: resolving bacterial genome assemblies from short and long sequencing reads. PLoS Comput Biol 13:e1005595. doi:10.1371/journal.pcbi.1005595 28594827 PMC5481147

[8] Wick RR, Judd LM, Gorrie CL, Holt KE. 2017. Completing bacterial genome assemblies with multiplex MinION sequencing. Microb Genom 3:e000132. doi:10.1099/mgen.0.000132 29177090 PMC5695209

[9] Deneke C, et al.. 2020. Milonga: a snakemake workflow for long-read assembly. Github. https://gitlab.com/bfr_bioinformatics/milonga.

[10] Deneke C. 2023. Bacterial characterization pipeline. Available from: https://gitlab.com/bfr_bioinformatics/bakcharak

[11] Seemann T. 2020. Abricate, Github. https://github.com/tseemann/abricate.

[12] Chen L, Zheng D, Liu B, Yang J, Jin Q. 2016. VFDB 2016: hierarchical and refined dataset for big data analysis--10 years on. Nucleic Acids Res 44:D694–7. doi:10.1093/nar/gkv1239 26578559 PMC4702877

[13] Seemann T. 2022. MLST Github. https://github.com/tseemann/mlst.

[14] Jolley KA, Maiden MCJ. 2010. BIGSdb: scalable analysis of bacterial genome variation at the population level. BMC Bioinformatics 11:595. doi:10.1186/1471-2105-11-595 21143983 PMC3004885

[15] Yoshida CE, Kruczkiewicz P, Laing CR, Lingohr EJ, Gannon VPJ, Nash JHE, Taboada EN. 2016. The Salmonella in silico typing resource (SISTR): an open web-accessible tool for rapidly typing and subtyping draft salmonella genome assemblies. PLoS One 11:e0147101. doi:10.1371/journal.pone.0147101 26800248 PMC4723315

[16] Folkesson A, Löfdahl S, Normark S. 2002. The Salmonella enterica subspecies I specific centisome 7 genomic Island encodes novel protein families present in bacteria living in close contact with eukaryotic cells. Res Microbiol 153:537–545. doi:10.1016/s0923-2508(02)01348-7 12437215

[17] Ray S, Pandey NK, Kushwaha GS, Das S, Ganguly AK, Vashi N, Kumar D, Suar M, Bhavesh NS. 2022. Structural investigation on SPI-6-associated Salmonella typhimurium VirG-like stress protein that promotes pathogen survival in macrophages. Protein Sci. 31:835–849. doi:10.1002/pro.4272 34997791 PMC8927873

[18] Deneke C, Uelze L, Brendebach H, Tausch SH, Malorny B. 2021. Decentralized investigation of bacterial outbreaks based on hashed cgMLST. Front Microbiol 12:649517. doi:10.3389/fmicb.2021.649517 34220740 PMC8244591

[19] Haft DH, DiCuccio M, Badretdin A, Brover V, Chetvernin V, O’Neill K, Li W, Chitsaz F, Derbyshire MK, Gonzales NR, Gwadz M, Lu F, Marchler GH, Song JS, Thanki N, Yamashita RA, Zheng C, Thibaud-Nissen F, Geer LY, Marchler-Bauer A, Pruitt KD. 2018. RefSeq: an update on prokaryotic genome annotation and curation. Nucleic Acids Res 46:D851–D860. doi:10.1093/nar/gkx1068 29112715 PMC5753331

[20] Li W, O’Neill KR, Haft DH, DiCuccio M, Chetvernin V, Badretdin A, Coulouris G, Chitsaz F, Derbyshire MK, Durkin AS, Gonzales NR, Gwadz M, Lanczycki CJ, Song JS, Thanki N, Wang J, Yamashita RA, Yang M, Zheng C, Marchler-Bauer A, Thibaud-Nissen F. 2021. RefSeq: expanding the prokaryotic genome annotation pipeline reach with protein family model curation. Nucleic Acids Res 49:D1020–D1028. doi:10.1093/nar/gkaa1105 33270901 PMC7779008

[21] Tatusova T, DiCuccio M, Badretdin A, Chetvernin V, Nawrocki EP, Zaslavsky L, Lomsadze A, Pruitt KD, Borodovsky M, Ostell J. 2016. NCBI prokaryotic genome annotation pipeline. Nucleic Acids Res 44:6614–6624. doi:10.1093/nar/gkw569 27342282 PMC5001611

[22] Liu B, Zheng D, Zhou S, Chen L, Yang J. 2022. VFDB 2022: a general classification scheme for bacterial virulence factors. Nucleic Acids Res 50:D912–D917. doi:10.1093/nar/gkab1107 34850947 PMC8728188

[23] Connor TR, Owen SV, Langridge G, Connell S, Nair S, Reuter S, Dallman TJ, Corander J, Tabing KC, Le Hello S, Fookes M, Doublet B, Zhou Z, Feltwell T, Ellington MJ, Herrera S, Gilmour M, Cloeckaert A, Achtman M, Parkhill J, Wain J, De Pinna E, Weill F-X, Peters T, Thomson N. 2016. What's in a name? species-wide whole-genome sequencing resolves invasive and noninvasive lineages of Salmonella enterica serotype Paratyphi B. mBio 7:e00527-16. doi:10.1128/mBio.00527-16 27555304 PMC4999539

[24] Deneke C, Brendebach H, Uelze L, Borowiak M, Malorny B, Tausch SH. 2021. Species-specific quality control, assembly and contamination detection in microbial isolate sequences with AQUAMIS. Genes (Basel) 12:644. doi:10.3390/genes12050644 33926025 PMC8145556

[25] Tatusova TA, Madden TL. 1999. BLAST 2 sequences, a new tool for comparing protein and nucleotide sequences. FEMS Microbiol Lett 174:247–250. doi:10.1111/j.1574-6968.1999.tb13575.x 10339815

